# Calibrating tetranucleotide-frequency distances for metagenomic binning with right-skewed distribution models

**DOI:** 10.1093/bioadv/vbag207

**Published:** 2026-07-25

**Authors:** Omar Hajjaji, Al-Sayed Al-Soudy, Rachid Daoud, Rachid Benhida, Morad M Mokhtar

**Affiliations:** College of Chemical Sciences and Engineering (CCSE), Chemical and Biochemical Sciences (CBS), Mohammed VI Polytechnic University (UM6P), Benguerir 43150, Morocco; College of Chemical Sciences and Engineering (CCSE), Chemical and Biochemical Sciences (CBS), Mohammed VI Polytechnic University (UM6P), Benguerir 43150, Morocco; College of Chemical Sciences and Engineering (CCSE), Chemical and Biochemical Sciences (CBS), Mohammed VI Polytechnic University (UM6P), Benguerir 43150, Morocco; College of Chemical Sciences and Engineering (CCSE), Chemical and Biochemical Sciences (CBS), Mohammed VI Polytechnic University (UM6P), Benguerir 43150, Morocco; Institut de Chimie de Nice, Université Côte d’Azur, Nice 06108, France; College of Chemical Sciences and Engineering (CCSE), Chemical and Biochemical Sciences (CBS), Mohammed VI Polytechnic University (UM6P), Benguerir 43150, Morocco

## Abstract

**Summary:**

Metagenomic binning is a pivotal step in reconstructing metagenome-assembled genomes (MAGs) from complex microbial communities, and it critically depends on reliable measures of similarity between contigs. In many workflows, tetranucleotide-frequency (TNF) distances are translated into probabilistic evidence of a shared genome of origin. Despite their central role, these distances are often modeled with convenient but poorly matched assumptions, even though they are intrinsically non-negative and frequently exhibit pronounced right-skewness—features that can distort tail behavior and weaken downstream thresholding decisions. In this work, we introduce a likelihood-based framework for characterizing intra- and inter-genomic TNF distance distributions with flexible right-skewed parametric models and for converting fitted distributions into calibrated distance-to-probability scores within a MaxBin-style scheme. Our approach provides a principled statistical basis for distributional assessment, probability calibration, and transparent operating-point selection, with the goal of improving robustness and interpretability in TNF-driven binning.

**Availability and implementation:**

All codes related to the article are available through a public GitHub repository at https://github.com/omar-hajjaji/Calibrating-TNF-Distances-for-Metagenomic-Binning-with-Right-Skewed-Distribution-Models.

## 1 Introduction

Microorganisms including bacteria, archaea, fungi, viruses, and protists are ubiquitous across natural environments. Diverse microbial communities and their genomes thrive within a wide range of habitats, from host-associated niches such as the human gut and plant rhizosphere (Scher and Abramson 2011, Walter and Ley 2011, Philippot *et al.* 2013) to extreme environments such as acid mine drainage (Simmons *et al.* 2008) and geothermal hot springs (Sharpton 2014).

Understanding microbial diversity is critical not only for conserving and maintaining ecosystems but also for advancing applications in agriculture, food science, biotechnology, animal and plant nutrition, and human health (Xia *et al.* 2023, Li *et al.* 2024). However, most microbial life is notoriously difficult to culture in the laboratory, complicating efforts to identify microbes and understand community-level functions. High-throughput sequencing technologies have transformed this challenge by enabling genomic analysis of all microbes in a sample, including those that cannot be cultured, thus providing a powerful approach for studying microbial communities in situ (Riesenfeld *et al.* 2004). One such method, shotgun metagenomics, is a comprehensive environmental sequencing strategy that analyses all genomic content present in a sample, offering insights into community biodiversity, metabolic potential, and ecological patterns (Sharpton 2014, Kellom *et al.* 2025). In particular, microbiome research has expanded rapidly in both data generation and methodological development since the release of the first shotgun metagenome from environmental samples (Venter *et al.* 2004).

Shotgun metagenomics, also known as short-read, second-generation, next-generation, or high-throughput sequencing, captures all DNA in a sample to recover whole-genome content. Unlike amplicon-based profiling methods that target specific marker genes such as 16S rRNA (Hamady and Knight 2009), shotgun metagenomics provides a relatively unbiased representation of the complete taxonomic composition and functional potential of microbial communities (Quince *et al.* 2017, Wang *et al.* 2023). Importantly, shotgun metagenomic sequencing also enables the reconstruction of metagenome-assembled genomes (MAGs), offering high-resolution insights into microbial diversity down to the species and often even strain level (Quince *et al.* 2017, Liu *et al.* 2021).

As metagenomic databases expand, meta-analyses can integrate data across thousands of heterogeneous datasets (Roux *et al.* 2019, Nayfach *et al.* 2021, Richardson *et al.* 2023). Thus, shotgun metagenomics has become a transformative tool in microbiome research, greatly improving our understanding of the structure and function of the microbial community in diverse environments (Nayfach *et al.* 2016, Guo *et al.* 2025).

A typical shotgun metagenomic workflow consists of five major stages: (i) sample collection, library preparation, and sequencing, (ii) computational preprocessing of reads (short- and/or long-read), including quality control (QC), (iii) taxonomic, functional, and genomic analyses using a combination of read-based, assembly-based, and binning approaches, (iv) statistical and biological post-processing, and (v) validation. Despite its apparent simplicity, shotgun metagenomics presents several challenges, including experimental biases, variability among analysis pipelines, and the complexity of computational, statistical, and visualization methods.

Within this workflow, metagenomic binning is a crucial downstream task that groups assembled contigs or long-read sequences originating from the same organism into bins in order to reconstruct MAGs. Binning methods exploit several complementary sources of information, including sequence-composition features such as GC content and short *k*-mer frequencies, differential abundance or coverage profiles, marker-gene information, linkage evidence, and, increasingly, assembly-graph structure (Sedlar *et al.* 2017, Mallawaarachchi *et al.* 2024). Obtaining robust sequence representations remains a major obstacle for improving binning accuracy (Fu *et al.* 2024). Genomes from different microbial species often contain characteristic nucleotide composition patterns, resulting in distinct short *k*-mer signatures, particularly tetranucleotide frequencies (TNFs). TNF is widely used because it is fast to compute, independent of assembly quality, and relatively stable across coverage variations (Karlin 1997, Teeling *et al.* 2004a, 2004b). Metrics derived from TNFs are among the most informative features of metagenomic binning (Dick *et al.* 2009). Their small size (k=4), universal presence across metagenomes, and phylogenetically informative usage patterns make them particularly effective for supervised learning and for resolving taxonomic relationships—often outperforming di- and trinucleotide measures (Noble *et al.* 1998, Pride *et al.* 2003, Teeling *et al.* 2004b, Bokulich 2025). The TNF vector has 136 components because each tetranucleotide is merged with its reverse complement, yielding a strand-invariant canonical representation. In this context, a natural measure of whether two fragments originate from the same genome is their genomic distance, representing the dissimilarity between their TNF vectors, commonly assessed using Euclidean distance. Small distances indicate intra-genomic similarity, whereas larger distances suggest inter-genomic separation. This TNF-based signal underpins many practical binning tools, including MaxBin/MaxBin2, MetaBAT2, CONCOCT, MetaDecoder, and graph-aware tools such as MetaCoAG, which combine composition, coverage, and assembly-graph information to recover MAGs from complex samples (Alneberg *et al.* 2014, Kang *et al.* 2019, Liu *et al.* 2022, Mallawaarachchi and Lin 2022).

Despite its centrality, the statistical characterization of TNF-based distances remains underdeveloped. In practice, pipelines often assume Gaussianity, apply fixed heuristics, or implicitly treat histograms as approximately normal when setting thresholds. For example, Gaussian mixture models are common for clustering multidimensional composition and coverage features (Alneberg *et al.* 2014). Yet pairwise distances are frequently right-skewed with heavier-than-Gaussian tails. Several mechanisms contribute to this shape: (i) finite sample variability in *k*-mer counts that decreases with fragment length, (ii) genome-level heterogeneity (e.g. GC content regimes, codon usage) that broadens the inter-genomic distribution, and (iii) technical artifacts (assembly breaks, chimeras) that introduce outliers. Empirically, normality-based rules can therefore miscalibrate tails, be overly conservative near genome boundaries, and overly aggressive in diverse communities, yielding suboptimal thresholds and downstream errors.

**Figure 1:**
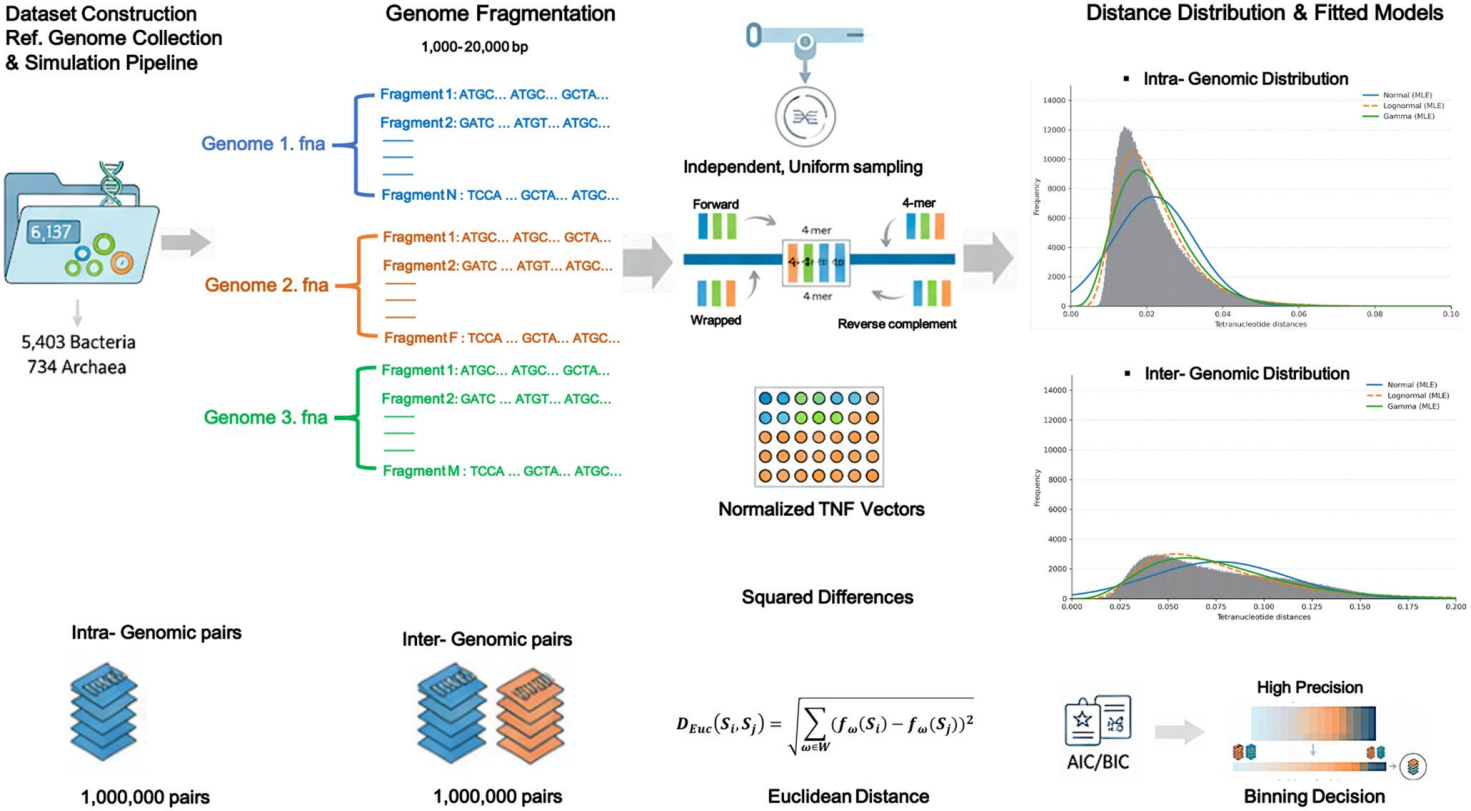
Workflow for TNF-based distance simulation and modeling. Overview of the dataset construction and simulation pipeline. A total of 6137 complete prokaryotic genomes were collected and converted into contig-like fragment catalogs, TNF vectors were computed using orientation-invariant canonical 4-mers, and Euclidean distances were calculated for 1M intra- and 1M inter-genomic fragment pairs. Empirical distance distributions were fitted with right-skewed parametric models (Gamma, Lognormal, Weibull, Beta) and evaluated using AIC/BIC to support calibrated metagenomic binning. The figure is displayed across the full page width to improve readability.

In this article, we develop a principled framework for modeling the empirical distributions of intra- and inter-genomic distances derived from TNF. We evaluate flexible parametric families appropriate for non-negative, right-skewed data (Beta, Gamma, Weibull, and Lognormal) by model comparison using the most widely used forms of model selection criteria, likelihood-based criteria, Bayesian Information Criterion (BIC), and Akaike Information Criterion (AIC) (Akaike 1974, Schwarz 1978), with validation using MaxBin2 methodology. Figure 1 summarizes the overall workflow, from genome collection and fragment-catalogue construction to TNF-distance computation, parametric model fitting, and AIC/BIC-based model selection.

## 2 Methods

### 2.1 Datasets access

A total of 6137 complete prokaryotic genomes (5403 Bacteria and 734 Archaea) were retrieved from the NCBI database (https://www.ncbi.nlm.nih.gov/datasets/genome/). The corresponding NCBI assembly accessions and organism names are provided in Files 1 (Bacteria) and 2 (Archaea), available as supplementary data at *Bioinformatics Advances* online. Genome selection was not explicitly stratified according to genomic %G+C content. However, plasmid sequences were excluded from the analysis to reduce additional compositional heterogeneity associated with extrachromosomal elements. We acknowledge that %G+C content can influence TNF-based distances, since tetranucleotide frequencies are partly constrained by the underlying nucleotide composition of each genome. Nevertheless, %G+C alone does not fully determine TNF profiles: genomes with similar %G+C may still differ in higher-order oligonucleotide usage, codon usage, and genome-signature patterns. Therefore, TNF provides a richer compositional representation than %G+C alone for characterizing intra- and inter-genomic distances.

For validation, benchmark datasets were used with known ground truth together with complementary real sequencing datasets. First, CAMI Toy low- and medium-complexity datasets were used as controlled benchmarks with known genome of origin assignments, allowing precision and sensitivity to be computed directly (Meyer *et al.* 2022). Second, the ATCC MSA-1003 mock-community dataset was used as a controlled real sequencing dataset with a defined microbial composition (ATCC 2026). Third, 10 Human Microbiome Project (HMP) metagenomes were used to evaluate performance across natural human-associated microbiomes spanning stool (SAMN00034120, SAMN00033491, SAMN00032708), saliva (SAMN00035910, SAMN00040338), tongue dorsum (SAMN00032552, SAMN00032577, SAMN46110235), and supragingival plaque (SAMN00032563, SAMN00032692). The per-sample BioSample accessions and corresponding SRA runs are listed in Table 3, available as supplementary data at *Bioinformatics Advances* online (Huttenhower *et al.* 2012). For all real sequencing datasets, reads were assembled using metaSPAdes (Nurk *et al.* 2017), and the Gaussian MaxBin2-like baseline and the Lognormal-based model were evaluated with the same preprocessing, assembly, contig filtering, binning, and CheckM2-based MAG-quality workflow (Chklovski *et al.* 2023).

### 2.2 Datasets simulation

The simulation was carried out in two distinct steps: fragment-catalog generation followed by Monte-Carlo sampling of pairwise comparisons. First, each collected genome was treated as circular and converted into a finite catalog of simulated contig-like fragments. Fragment lengths *L* were drawn uniformly from 1000 to 20 000 bp, and fragments were extracted sequentially along each genome until the genome sequence had been covered. The number of fragments was therefore not fixed a priori, but depended on genome length and on the random fragment lengths drawn during this fragmentation step. Pair generation was performed from the precomputed fragment catalog of each genome rather than by selecting replicons during pair sampling.

The upper bound of 20 000 bp was chosen to focus on a contig-scale regime that is directly relevant to metagenomic binning after short-read assembly. Very long fragments, approaching hundreds of kilobases or megabase scale, provide more stable tetranucleotide-frequency (TNF) estimates and may dominate the fitted distributions with distances that are less representative of the ambiguous contig-level cases encountered in practical binning.

Second, the reported 1 000 000 intra-genomic and 1 000 000 inter-genomic observations refer to pairwise TNF-distance observations, not to one pair per genome. For each intra-genomic observation, one genome was selected uniformly from the 6137 genomes and two distinct fragments were sampled from the fragment catalog of that genome. This procedure was repeated until 1 000 000 intra-genomic comparisons were obtained. For each inter-genomic observation, two distinct genomes were selected uniformly and one fragment was sampled from the catalog of each selected genome. This procedure was repeated until 1 000 000 inter-genomic comparisons were obtained. Uniform sampling across genomes was used to prevent overrepresentation of longer genomes. Replacement was allowed across Monte-Carlo iterations because the objective was not to simulate a non-redundant metagenomic assembly with a fixed abundance structure, but to estimate empirical class-conditional distributions of TNF-based Euclidean distances for random intra- and inter-genomic fragment comparisons. Consequently, the same fragment could contribute to more than one pairwise comparison and could appear in both the intra- and inter-genomic sampling sets. However, within a single intra-genomic comparison the same fragment was not paired with itself, and within a single inter-genomic comparison the two fragments were required to originate from different genomes.

**Figure 2:**
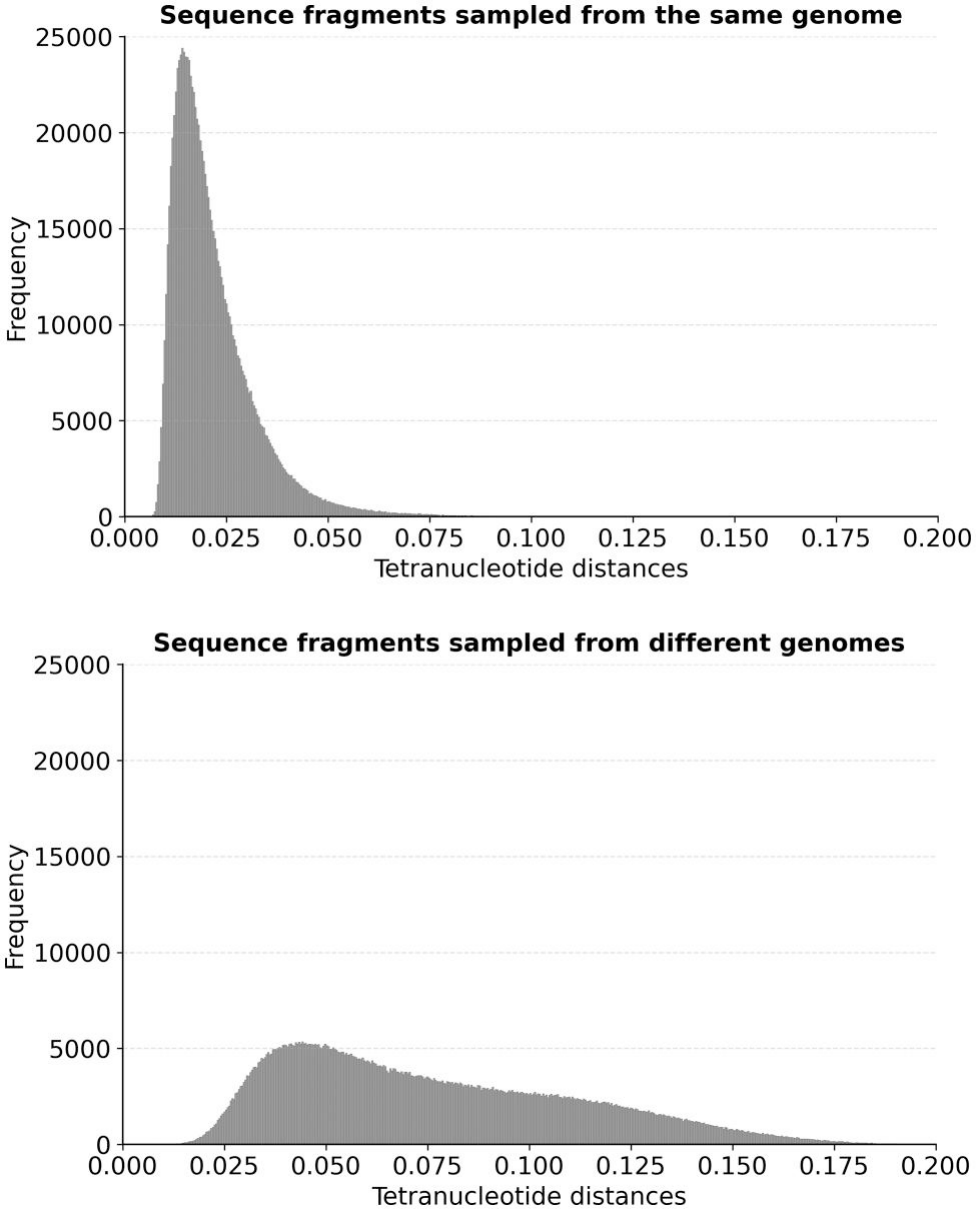
Distributions of genomic distances based on tetranucleotide frequencies (TNF). Top, intra-genomic distances; bottom, inter-genomic distances. The vertical arrangement improves readability of the two empirical distributions.

Inter-genomic sampling was not explicitly stratified by taxonomic distance. Therefore, the inter-genomic distribution shown in Figure 2 should be interpreted as a dataset-dependent empirical background distribution of TNF distances between fragments originating from different genomes, rather than as a universal inter-genome distribution. Because the two genomes in each inter-genomic pair were sampled only under the constraint of being distinct, the resulting distribution may include both relatively close comparisons, such as genomes sharing a recent common ancestor at the genus or family level, and more distant comparisons, such as order-level or higher relationships. Consequently, the observed shape of the inter-genomic distribution may reflect a mixture of taxonomic-distance regimes and may depend on the relative number of close and distant genome pairs present in the selected genome set.

### 2.3 Tetranucleotide frequencies and Euclidean distance

For each fragment *S* with length *L*, we computed a strand-invariant TNF vector by scanning the sequence with a 1 bp sliding window and counting all overlapping 4-mers on the forward strand and on the reverse complement where windows containing non-{A, C, G, T} symbols were skipped. To ensure orientation invariance, each 4-mer was merged with its reverse complement, yielding a canonical set of 136 distinct tetramers where palindromes were counted just once (Pride *et al.* 2003, Teeling *et al.* 2004a). Let W denote these 136 tetramers and $c_{w}(S)$ the count of windows whose canonical 4-mer is w∈W. We define the length-normalized frequency vector $f(S)\in R_{+}^{136}$ by


$$f_{w}(S)=\frac{c_{w}(S)}{\sum_{u\in W}c_{u}(S)},\;\sum_{w\in W}f_{w}(S)=1.$$

Pairwise compositional dissimilarity between fragments $S_{i}$ and $S_{j}$ was then measured as the Euclidean distance between their TNF vectors:


$$D_{\text{Euc}}(S_{i},S_{j})={\left\|f(S_{i})-f(S_{j})\right\|}_{2}=\sqrt{\sum_{w\in W}{\left(f_{w}(S_{i})-f_{w}(S_{j})\right)}^{2}}.$$

In the simulations, the resulting TNF-based Euclidean distances were empirically confined to the interval (0,1) for both intra- and inter-genomic pairs. We therefore treated *D* as a bounded random variable in what follows and included bounded-support models when appropriate. This strand-merged, frequency normalized TNF representation and Euclidean distance are standard in the genome signature literature and orientation-independent of fragment composition (Pride *et al.* 2003, Teeling *et al.* 2004a).

As described above, the inter-genomic distribution in Figure 2 corresponds to the empirical mixture induced by the unstratified sampling of distinct genome pairs. The shapes in Figure 2 naturally call for right-skewed, non-negative models, and we fixed the location parameter on value 0. We therefore fit each class with parametric families whose support and tail behavior match the data: (i) Gamma(k,θ), a flexible non-negative family that ranges from a sharp mode near zero when k<1 to a unimodal, right-skewed profile for k>1 (Johnson *et al.* 1994), (ii) Weibull(λ,κ), which can place the mode away from zero for κ>1 and accommodates heavier right tails than the Gaussian (Weibull 1951, Johnson *et al.* 1994), and (iii) Lognormal(s,σ), appropriate when log(D) is approximately normal, yielding a long right tail well suited to the inter-genomic shoulder (Johnson *et al.* 1994, Limpert *et al.* 2001). Since the empirical distances in the simulations satisfy 0<D<1, we also include the Beta(a,b) family, which respects this observed bounded support and avoids allocating probability mass outside the feasible range, moderate (a,b) values capture the intra-class peak near zero with a thinning tail (a<1≤b) and the inter-class unimodality away from zero with asymmetric spread (a>1,b>1) (Johnson *et al.* 1994).

### 2.4 Goodness-of-fit

All candidate models (Gamma, Weibull, Lognormal, and Beta) are fitted by maximum likelihood estimation (MLE) directly on the observed distances *D*, and relative fit is assessed with Akaike’s and Bayesian information criteria (AIC/BIC) (Akaike 1974, Schwarz 1978). Because the empirical distributions are bounded and right-skewed, we expect at least one of these asymmetric families to capture both the peak and upper-tail behavior more faithfully than a symmetric Gaussian, whose unbounded support and symmetry typically overspread mass near the center and underestimate the right tail.

We deliberately use information criteria rather than goodness-of-fit tests. With sample sizes of order $10^{6}$ per class, Empirical Distribution Function–based tests (Kolmogorov–Smirnov, Anderson–Darling) have overwhelming power, so even negligible discrepancies from any simple parametric form yield vanishing *P* values; such tests answer whether a model is exactly true, rather than whether it is the most useful approximation (D’Agostino and Stephens 1986, Wasserstein and Lazar 2016). Moreover, since the true generative law is unlikely to lie within our finite candidate set, the principled target is predictive adequacy under model misspecification (White 1982). We therefore rank models by AIC and BIC: AIC is an asymptotically unbiased estimator of out-of-sample Kullback–Leibler risk (Akaike 1974, Konishi and Kitagawa 2008), while BIC is consistent for selecting the simplest sufficient model when a true finite-dimensional model exists (Schwarz 1978, Claeskens and Hjort 2008).

In the results, we report for each class the maximized log-likelihood together with AIC/BIC and the differences relative to the class-best model (ΔAIC/ΔBIC); when a single family is desired across tasks, we also provide total criteria obtained by summation across classes, ${\text{AIC}}_{\text{total}}={\text{AIC}}_{\text{intra}}+{\text{AIC}}_{\;\text{inter}}$ (and analogously for BIC), to judge unified choices. To connect goodness-of-fit to binning decisions, we keep the same distance-to-probability mapping used in prior work (Wu *et al.* 2016). For an observed distance $d=\text{dist}(S_{1},S_{2})$,


(1) $$P_{\text{dist}}(S_{1}\in G(S_{2}))=\frac{N\left(d|\mu_{\text{intra}},\sigma_{\text{intra}}^{2}\right)}{N\left(d|\mu_{\text{intra}},\sigma_{\text{intra}}^{2}\right)+N\left(d|\mu_{\text{inter}},\sigma_{\text{inter}}^{2}\right)}.$$

where $N_{\text{intra}}$ and $N_{\text{inter}}$ are Gaussian densities with MLE parameters. We then compute the analogous mapping induced by whichever right-skewed family AIC/BIC selects, simply replacing the Gaussian densities by the fitted class-conditional densities,


(2) $$P_{\text{dist}}^{⋆}(S_{1}\in G(S_{2}))=\frac{f_{\text{intra}}\left(d|{\hat{\theta }}_{\text{intra}}\right)}{f_{\text{intra}}\left(d|{\hat{\theta }}_{\text{intra}}\right)+f_{\text{inter}}\left(d|{\hat{\theta }}_{\text{inter}}\right)}.$$

with $f_{\text{intra}}$ and $f_{\text{inter}}$ denoting the selected right-skewed densities and ${\hat{\theta }}_{\text{intra}},{\hat{\theta }}_{\text{inter}}$ their MLEs. Equations (1) and (2) share the same form, so improvements in tail fit translate directly into more accurate probabilities and, consequently, higher-precision binning at comparable recall.

## 3 Results

In this section, we evaluated how well candidate right-skewed, non-negative distributions describe the genomic distances derived from TNF vectors. For each class, we fitted Gamma, Weibull, Lognormal, and Beta models by maximum likelihood on the same sample of $N=10^{6}$ distances, alongside a Gaussian baseline for reference. The resulting maximum-likelihood parameter estimates for each family and each class are reported in Table 1. Model adequacy was summarized by the maximized log-likelihood and ranked using Akaike (AIC) and Bayesian (BIC) information criteria. Lower values indicate better expected out of sample fit, with differences ΔAIC>10 or ΔBIC>10 regarded as strong evidence.

**Table 1:** Parameter estimates for intra- and inter-genomic distance models.^a^

	Intra-distances		Inter-distances	
Distribution	Parameter 1	Parameter 2	Parameter 1	Parameter 2
Gamma	k=5.4306	θ=0.0040	k=4.5477	θ=0.0167
Weibull	λ=2.1340	κ=0.0246	λ=2.2927	κ=0.0864
Lognormal	s=0.4203	σ=0.0198	s=0.4881	σ=0.0680
Beta	a=5.2826	b=237.0206	a=4.2191	b=51.0936
Gaussian	μ=0.0217	σ=0.0107	μ=0.0762	σ=0.0357

a Parameters are maximum-likelihood estimates obtained with the location parameter fixed at zero. Gamma is parameterized by shape k and scale θ. For the Weibull, λ is the shape parameter and κ the scale parameter. For the lognormal, s denotes the shape parameter (log-scale standard deviation) and σ the estimated scale parameter, following the parameterization used in the implementation. Beta is parameterized by shape parameters a and b. Gaussian reports mean μ and standard deviation σ.

Across the four right-skewed families and the Gaussian baseline, AIC/BIC rankings are consistent within each class (Tables 2 and 3). For inter-genomic distances, the Gamma model attains the best score (AIC =−3 978 633.64; BIC =−3 978 610.01), with Beta a distant second (AIC difference ΔAIC=308.87) and Lognormal further behind (ΔAIC=7 408.60). Weibull and the Gaussian baseline are decisively worse (ΔAIC=40 586.22 and 153 642.78, respectively). Since lower AIC/BIC indicates better expected out-of-sample performance, these gaps provide very strong evidence in favor of Gamma for the unstratified inter-genomic background, which was not stratified by taxonomic distance and is expected to be dominated by relatively distant genome pairs.

**Table 2:** Comparison of AIC/BIC criteria between models for inter-distances.^a^

Distribution	Loglikelihood-inter	AIC-inter	BIC-inter
Gamma	**1 989 318.82**	**−3 978 633.64**	**−3 978 610.01**
Weibull	1 969 025.71	−3 938 047.42	−3 938 023.79
Lognormal	1 985 614.52	−3 971 225.04	−3 971 201.41
Beta	1 989 164.38	−3 978 324.77	−3 978 301.14
Gaussian	1 912 497.43	−3 824 990.86	−3 824 967.23

a Bold values indicate the best-fitting model, that is, the model with the highest log-likelihood and the lowest AIC and BIC. Loglikelihood-inter, AIC-inter and BIC-inter refer to the fits obtained on inter-genomic distances.

**Table 3:** Comparison of AIC/BIC criteria between models for intra-distances.^a^

Distribution	Loglikelihood-intra	AIC-intra	BIC-intra
Gamma	3 317 780.13	−6 635 556.27	−6 635 532.64
Weibull	3 212 598.17	−6 425 192.35	−6 425 168.72
Lognormal	**3 368 942.94**	−**6 737 881.89**	−**6 737 858.25**
Beta	3 314 169.98	−6 628 335.96	−6 628 312.33
Gaussian	3 117 429.34	−6 234 854.68	−6 234 831.05

a Bold values indicate the best-fitting model, that is, the model with the highest log-likelihood and the lowest AIC and BIC. Loglikelihood-intra, AIC-intra and BIC-intra refer to the fits obtained on intra-genomic distances.

For intra-genomic distances, the ranking reverses. Lognormal is best (AIC =−6 737 881.89; BIC =−6 737 858.25) by a wide margin over Gamma (ΔAIC=102 325.62) and Beta (ΔAIC=109 545.93), with Weibull and Gaussian far behind (ΔAIC=312 689.54 and 503 027.21). Because the candidate families have comparable dimensionality (two parameters with fixed location for Gamma/Weibull/Lognormal/Beta, two for Gaussian), these differences are driven by fit quality rather than complexity penalties.

Because some applications may prefer a single family across both classes, we also report Total-AIC/BIC in Table 4, obtained by summing criteria over inter and intra fits. Aggregating evidence across classes in Table 4, Lognormal yields the best overall criterion (Total-AIC =−10 709 106.93), reflecting its dominant advantage for intra-distances, Gamma is next (Total ΔAIC=94 917.01), followed by Beta (Total ΔAIC=102 446.19).

**Table 4:** Comparison of total AIC/BIC criteria between models for intra and inter distances.^a^

Distribution	Total-AIC	Total-BIC
Gamma	−10 614 189.92	−10 614 142.65
Weibull	−10 363 239.77	−10 363 192.51
Lognormal	−**10 709 106.93**	−**10 709 059.67**
Beta	−10 606 660.74	−10 606 613.47
Gaussian	−10 059 845.55	−10 059 798.28

a Bold values indicate the best unified model, that is, the model with the lowest total AIC and BIC. Total-AIC and Total-BIC are obtained by summing the criteria over the intra- and inter-genomic fits.

In sum, right-skewed families are strongly preferred over the Gaussian baseline. A class-specific description is supported for the simulated dataset, with Lognormal best fitting intra-genomic distances and Gamma best fitting the unstratified inter-genomic background dominated by longer evolutionary distances. If a single family must be used for both classes, Lognormal minimizes the total information criterion and provides a practical unified right-skewed model for downstream probability calibration.

**Figure 3:**
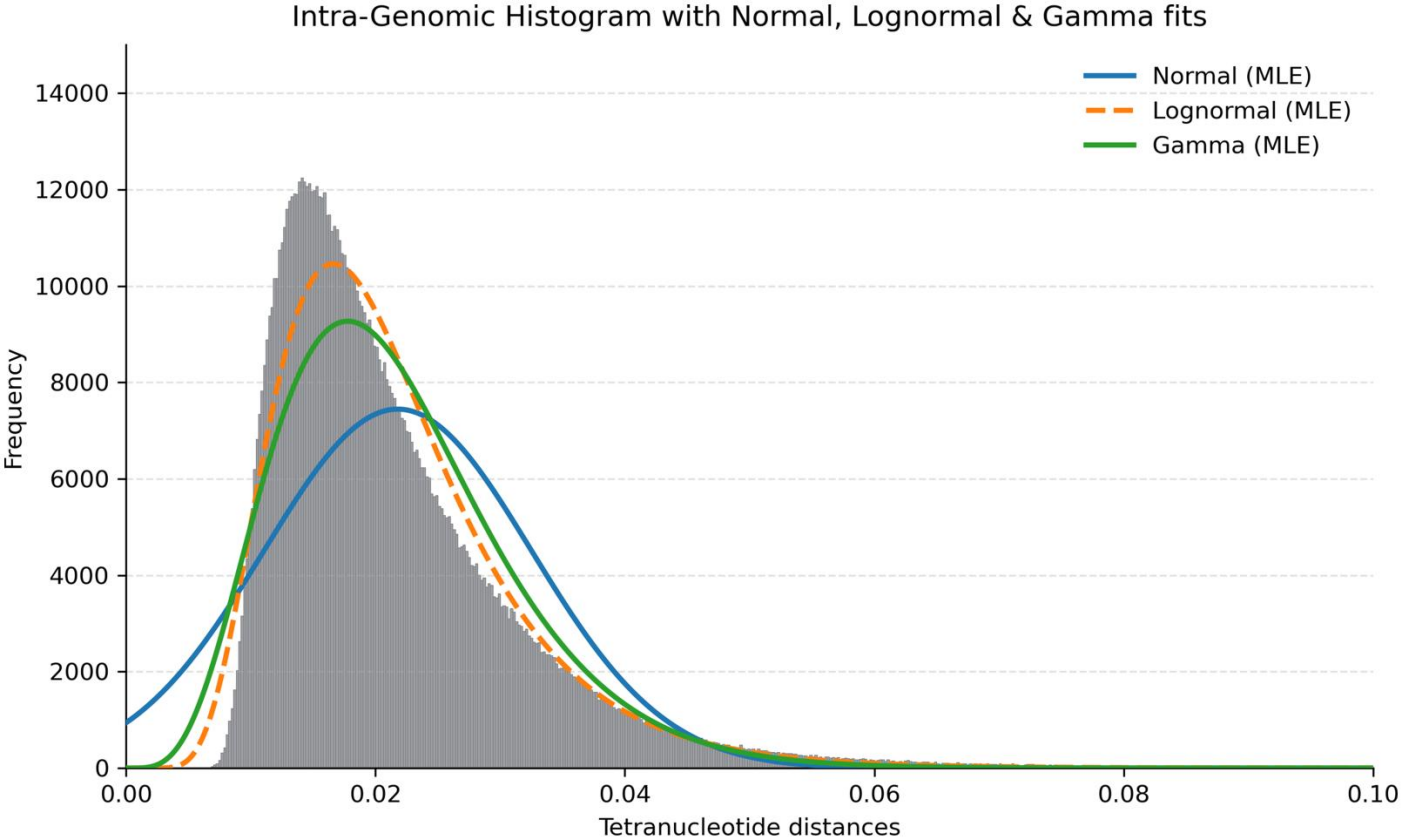
Intra-genomic distances. Histogram with overlaid MLE Gaussian, Gamma, and Lognormal densities.

**Figure 4:**
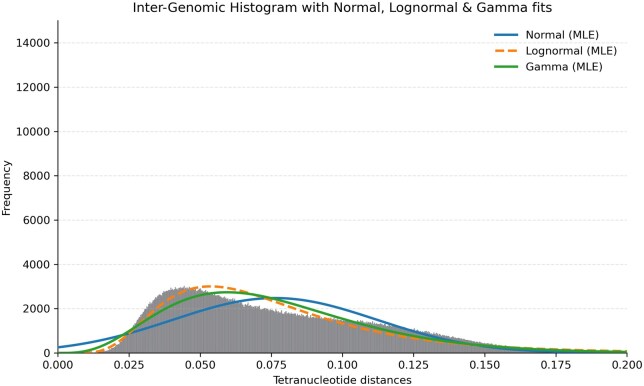
Inter-genomic distances. Histogram with overlaid MLE Gaussian, Gamma, and Lognormal densities.

To visualize absolute adequacy in the data space, Figures 3 and 4 overlay maximum-likelihood Gaussian, Lognormal, and Gamma densities on the empirical histograms for the intra- and inter-genomic distances, respectively. For the intra class, the Lognormal curve closely tracks the sharp peak at small distances (≈0.015−0.02) and the gradual right tail, whereas the Gaussian by symmetry overspreads mass around the center and underestimates the upper tail. The Gamma fit follows the peak location reasonably well but decays too quickly in the far right tail compared with the data, consistent with its higher AIC/BIC relative to Lognormal in Table 3.

For the unstratified inter-genomic background, the Gamma density reproduces the pronounced right skew with a mode near 0.05 and a slowly decreasing tail, aligning closely with the observed shoulder and tail behavior. The Lognormal captures the broad shape but slightly underestimates mass in the right tail beyond 0.10, while the Gaussian remains too diffuse, placing undue weight in the left tail and around the center. These visual patterns agree with the information-criterion rankings (Tables 2 and 3): right-skewed families substantially outperform a Gaussian baseline, with Lognormal preferred for intra-genomic distances and Gamma preferred for the unstratified inter-genomic background. In general, closely related inter-genomic pairs may produce distributions closer to the intra-genomic regime, for which a Lognormal model remains a reasonable right-skewed alternative.

### 3.1 Validation

To validate the practical impact of the proposed distributional modeling of TNF-based distances, we re-implemented the MaxBin2 scoring scheme described in Wu *et al.* (2014, 2016) and evaluated whether replacing the Gaussian distance model by a Lognormal model improves downstream binning quality.

We performed a controlled comparison between two MaxBin2-like variants that differ only in the parametric model used to map TNF distances to probabilities: (i) the original Gaussian-based formulation in Equation (1) and (ii) the Lognormal-based formulation in Equation (2). All other components, including TNF-distance computation, preprocessing strategy, assembly, binning decision rules, and evaluation protocol, were kept identical within each dataset so that observed differences could be attributed to the assumed distance distribution.

We evaluated both variants on CAMI Toy datasets, where the ground truth genome-of-origin is known. Following MaxBin (Wu *et al.* 2014), we summarized binning quality using precision (bin purity) and sensitivity (genome recovery), computed from the contingency table $R_{ij}$, where $R_{ij}$ denotes the amount of sequence from reference genome *j* assigned to predicted bin *i* (in base pairs). The metrics are defined as:


$$\begin{array}{c} \text{Precision}=\frac{\sum_{i=1}^{M}{\text{max}}_{j}R_{ij}}{\sum_{i=1}^{M}\sum_{j=1}^{N}{\text{max}}_{j}R_{ij}},\\ \text{Sensitivity}=\frac{\sum_{j=1}^{N}{\text{max}}_{i}R_{ij}}{\sum_{i=1}^{M}\sum_{j=1}^{N}{\text{max}}_{j}R_{ij}+\text{unclassified}\;\text{sequences}} \end{array}$$

In addition to the CAMI ground-truth validation, we assessed the two models on real sequencing datasets using CheckM2 completeness and contamination estimates. The real-data evaluation was organized into two complementary parts. First, the ATCC MSA-1003 dataset was used as a real mock-community benchmark with a defined microbial composition. Second, 10 HMP metagenomes were used to assess performance across natural human-associated microbiomes. For each dataset, the Gaussian MaxBin2-like baseline and the Lognormal-based model were evaluated under an identical workflow. HMP results are summarized in the main text as mean and standard deviation across samples, and the full per-sample CheckM2 results are provided in Table S3, available as supplementary data at *Bioinformatics Advances* online.

As reported in Table 5, the Lognormal-based variant consistently improves both precision and sensitivity over the Gaussian-based variant on CAMI_TOY_low and CAMI_TOY _medium. This empirical gain supports our modeling choice: by better capturing the right-skewed behavior of TNF distance distributions, the Lognormal mapping provides a more discriminative probability calibration in the overlap region between intra- and inter-genomic distances, which translates into bins that are simultaneously purer and more complete.

**Table 5:** Comparison of binning performance (precision and sensitivity) between Lognormal- and Gaussian-based MaxBin2-like models on CAMI Toy datasets.^a^

Dataset	Model	Precision	Sensitivity
CAMI_TOY_low	MaxBin2-Lognormal	**0.9008**	**0.8279**
	MaxBin2-Gaussian	0.8381	0.7557
CAMI_TOY_medium	MaxBin2-Lognormal	**0.7063**	**0.7806**
	MaxBin2-Gaussian	0.6287	0.6714

a Bold values indicate the best performance within each dataset and metric.

The real-data results further support this conclusion while providing a complementary assessment based on MAG quality estimates. On the ATCC MSA-1003 real mock-community dataset (Table 6), the Lognormal-based model recovered fewer bins than the Gaussian model (11 vs. 12), but achieved higher average completeness, lower average and median contamination, and a larger number of medium-quality-or-better bins. Across the 10 HMP real metagenomes (Table 7), the Lognormal-based model produced slightly fewer bins on average but increased mean completeness by 4.42 ± 3.39 percentage points and increased the number of medium-quality-or-better bins by 1.10 ± 2.23 bins relative to the Gaussian baseline. Contamination estimates remained broadly comparable between the two models, with only minor average differences (+0.64 ± 1.23 percentage points for mean contamination and +0.49 ± 2.93 percentage points for median contamination). In addition, median contamination was lower with the Lognormal model in 7 out of 10 HMP samples.

**Table 6:** MAG quality comparison on the ATCC MSA-1003 real mock-community dataset using CheckM2 estimates.^a^

Model	Bins	Mean comp. (%)	Mean contam. (%)	Median contam.	HQ bins	MQ/HQ bins
Gaussian	12	68.97	8.58	7.37	**2**	4
Lognormal	11	**76.79**	**7.86**	**3.45**	**2**	**5**

a HQ bins denote high-quality bins, defined as completeness ≥90% and contamination ≤5%. MQ/HQ bins denote medium-quality or better bins, defined as completeness ≥50% and contamination ≤10%. Bold values indicate the best value within each metric.

**Table 7:** MAG quality summary across 10 HMP real metagenomes using CheckM2 estimates.^a^

Metric	Gaussian baseline	Lognormal model	Change
	Mean ± SD	Mean ± SD	Lognormal − Gaussian
No. bins	18.90 ± 9.88	17.40 ± 9.48	−1.50 ± 1.18
Mean completeness (%)	57.32 ± 9.97	61.74 ± 10.86	+4.42 ± 3.39
Mean contamination (%)	10.78 ± 2.67	11.41 ± 3.23	+0.64 ± 1.23
Median contamination (%)	8.38 ± 2.49	8.87 ± 3.01	+0.49 ± 2.93
HQ bins	0.50 ± 0.71	0.70 ± 0.82	+0.20 ± 0.42
MQ/HQ bins	3.90 ± 2.96	5.00 ± 3.23	+1.10 ± 2.23

a Values are reported as mean  ±  standard deviation across samples. The last column reports the paired difference Lognormal − Gaussian. HQ bins denote high-quality bins, defined as completeness ≥90% and contamination ≤5%. MQ/HQ bins denote medium-quality or better bins, defined as completeness ≥50% and contamination ≤10%.

## 4 Discussion

TNF-based Euclidean distances are central to composition-driven metagenomic binning, yet their statistical structure is often simplified for convenience. In the simulations, both intra- and inter-genomic TNF distances exhibited pronounced right-skewness, making the Gaussian assumption poorly matched to the empirical distributions. In contrast, right-skewed parametric families provided substantially better empirical fidelity. Information-theoretic model selection supported a class-specific description for the simulated dataset, with Lognormal best fitting intra-genomic distances and Gamma best fitting the unstratified inter-genomic background dominated by longer evolutionary distances.

To characterize the taxonomic structure underlying this background, we determined the lowest common ancestor (LCA) of every inter-genomic pair from the taxonomic labels of the two sampled genomes. Same-genus comparisons constitute only 0.33% of the pairs, the remaining 99.67% being different-genus comparisons whose LCA lies at the family level or above, of which approximately one-fifth (21.2%) are cross-domain Bacteria–Archaea pairs (Table 4, available as supplementary data at *Bioinformatics Advances* online). The unstratified background is thus overwhelmingly composed of distant evolutionary comparisons, which accounts for its right-skewed, Gamma-favored shape. When the same-genus pairs are examined separately, however, their distances contract toward the intra-genomic regime (mean 0.029, median 0.026, against an intra-genomic mean of approximately 0.022 and an overall inter-genomic mean of 0.076), and a Lognormal model describes this subset better than a Gamma (ΔAIC=244). The Gamma fit therefore reflects the long evolutionary distance regime that dominates the unstratified background rather than short, genus-level distances, whereas a right-skewed model and the Lognormal in particular remains preferable to the Gaussian across both regimes (Akaike 1974, Schwarz 1978, Burnham and Anderson 2002).

The practical consequence of adopting distribution-aware models is not merely improved fit, but improved calibration. Rather than applying heuristic or symmetric cutoffs directly to TNF distances, each observed distance can be mapped to evidence of shared genome-of-origin using class-conditional densities, for example through a likelihood ratio or posterior probability. This calibration is particularly important in the overlap region between the right tail of the intra-genomic distribution and the left shoulder of the inter-genomic distribution, where binning errors are most likely to occur. In this region, Gaussian models tend to misrepresent tail behavior, whereas right-skewed models provide a more appropriate representation of the empirical distance structure.

The validation experiments support this interpretation. On the CAMI Toy datasets, where contig-level ground truth is available, the Lognormal-based MaxBin2-like variant improved both precision and sensitivity compared with the Gaussian baseline. The real-data validation provides complementary evidence under more practical conditions. On the ATCC MSA-1003 real mock-community dataset, the Lognormal-based model produced fewer bins but achieved higher average completeness, lower average and median contamination, and more medium-quality-or-better bins (Table 6). Across 10 HMP real metagenomes, the Lognormal-based model increased mean completeness and the number of medium-quality-or-better bins relative to the Gaussian baseline, while producing slightly fewer bins on average (Table 7). Contamination estimates remained broadly comparable between the two models, and median contamination was lower with the Lognormal model in most HMP samples. These results indicate a favorable and reproducible MAG-quality trend across controlled and natural metagenomic datasets, while still recognizing that individual quality metrics may remain sample dependent.

Several limitations should be noted. First, the inter-genomic distribution used in this study was generated by unstratified sampling of distinct genome pairs. Because the sampled inter-genomic pairs are dominated by relatively distant comparisons, the Gamma fit observed for the inter-genomic background mainly describes longer evolutionary distances. As quantified above (Table 4, available as supplementary data at *Bioinformatics Advances* online), same-genus comparisons account for only a small fraction of the pairs and their distances shift toward the intra-genomic regime, where a Lognormal model is preferred; a taxonomy-stratified background would therefore be expected to favor the Lognormal for both classes. Second, exact contig-level genome-of-origin labels were not available for the real HMP samples. Consequently, MAG quality was assessed using CheckM2 completeness and contamination estimates rather than direct ground-truth assignments (Chklovski *et al.* 2023).

Importantly, incorporating these models into existing workflows can be achieved with minimal disruption. A practical integration consists of: (i) computing TNF distances as usual, (ii) optionally stratifying by fragment length to reflect the length–variance relationship, (iii) fitting the chosen right-skewed model or models by maximum likelihood on the dataset at hand, and (iv) replacing the original distance-to-score mapping with a distribution-based likelihood ratio or posterior. Such calibrated scores can be used not only in MaxBin2-like probability calculations, but also as edge weights in contig graphs, or fused with coverage, marker-gene, and linkage evidence in modern binning workflows (Wu *et al.* 2016, Liu *et al.* 2022, Mallawaarachchi and Lin 2022). For assembly-graph-based approaches such as MetaCoAG, calibrated TNF probabilities could act as composition-based compatibility scores on graph edges, helping to down-weight weak or misleading connections and improving robustness against false edges during bin refinement.

Overall, these results support replacing Gaussian assumptions with right-skewed parametric models as a more appropriate calibration strategy for TNF-based distances. The proposed framework turns TNF distances into more interpretable and better calibrated evidence for genome-of-origin assignment, with improvements observed in controlled benchmarks and favorable MAG-quality trends in real metagenomic datasets.

## 5 Conclusion

In summary, TNF-based Euclidean distances are markedly right-skewed, so the Gaussian assumption commonly adopted in binning pipelines is systematically misspecified. Across large-scale simulations, information-criterion comparisons consistently favor right-skewed families over the Gaussian, with the Lognormal providing the strongest unified model when a single distribution must be used without prior knowledge of class labels. This improved tail calibration yields more reliable distance-to-probability mappings and better binning outcomes, as confirmed on both controlled benchmarks and real metagenomic datasets. More broadly, these results establish distribution-aware calibration as a principled alternative to fixed Gaussian or heuristic cutoffs for composition-based binning. Because the proposed mapping is a drop-in replacement for the distance-to-probability step, it can be integrated into existing tools with minimal change and extended in several directions. A first direction is length- or taxonomy-aware calibration that adapts the fitted distribution to fragment length and evolutionary distance. Beyond the integration scenarios detailed above, the same likelihood-based framework could further be applied to other compositional distances and *k*-mer representations, offering a general route toward better calibrated and more interpretable evidence for genome-of-origin assignment.

## Supplementary Material

vbag207_Supplementary_Data

## Data Availability

All relevant data are provided within the article and supplementary files. The scripts supporting this study are available through a public GitHub repository at https://github.com/omar-hajjaji/Calibrating-TNF-Distances-for-Metagenomic-Binning-with-Right-Skewed-Distribution-Models. A Zenodo archive is also available for peer review and long-term preservation at https://zenodo.org/records/17974314. Key points In the literature, genomic distances are often modeled under a Gaussian assumption; however, this approach is inadequate because it does not accurately capture the shape of the distance distribution. This study focuses on genomic distances and proposes a principled framework for modeling the distributions of intra- and inter-genomic distances. We evaluate flexible, non-Gaussian families appropriate for non-negative, right-skewed data, including Beta, Gamma, Weibull, and Lognormal distributions. We propose that using a Lognormal distribution instead of a Gaussian distribution improves probability estimation in binning tools such as MaxBin2, particularly in the step where probabilities are calculated to decide whether two sequences belong to the same bin.

## References

[vbag207-B1] Akaike H. A new look at the statistical model identification. IEEE Trans Automat Contr 1974;19:716–23. 10.1109/TAC.1974.1100705

[vbag207-B2] Alneberg J , Bjarnason BS, de Bruijn I et al Binning metagenomic contigs by coverage and composition. Nat Methods 2014;11:1144–6. 10.1038/nmeth.3103 25218180

[vbag207-B3] American Type Culture Collection (ATCC). 20 Strain Staggered Mix Genomic Material (MSA-1003). Manassas, VA: ATCC Microbiome Standards, 2026. https://www.atcc.org/products/msa-1003 (22 July 2026, date last accessed).

[vbag207-B4] Bokulich NA. Integrating sequence composition information into microbial diversity analyses with k-mer frequency counting. mSystems 2025;10:e01550-24. 39976436 10.1128/msystems.01550-24 PMC11915819

[vbag207-B5] Burnham KP , Anderson DR. Model Selection and Multimodel Inference: A Practical Information-Theoretic Approach. 2nd edn. New York: Springer, 2002.

[vbag207-B6] Chklovski A , Parks DH, Woodcroft BJ et al CheckM2: a rapid, scalable and accurate tool for assessing microbial genome quality using machine learning. Nat Methods 2023;20:1203–12. 10.1038/s41592-023-01940-w 37500759

[vbag207-B7] Claeskens G , Hjort NL. Model Selection and Model Averaging. Cambridge: Cambridge University Press, 2008.

[vbag207-B8] D’Agostino RB , Stephens MA. Goodness-of-Fit Techniques. New York: Marcel Dekker, 1986.

[vbag207-B9] Dick GJ , Andersson AF, Baker BJ et al Community-wide analysis of microbial genome sequence signatures. Genome Biology 2009;10:R85. 19698104 10.1186/gb-2009-10-8-r85 PMC2745766

[vbag207-B10] Fu L , Shi J, Huang B. Binning metagenomic contigs using contig embedding and decomposed tetranucleotide frequency. Biology (Basel) 2024;13:755. 39452065 10.3390/biology13100755 PMC11505167

[vbag207-B11] Guo P , Li C, Liu J et al Contribution of environmental and biological factors to bacterial community structure and stability in a subalpine lake. Mar Life Sci Technol 2025;7:176–86. 40027328 10.1007/s42995-024-00256-8 PMC11871254

[vbag207-B12] Hamady M , Knight R. Microbial community profiling for human microbiome projects: tools, techniques, and challenges. Genome Res 2009;19:1141–52. 19383763 10.1101/gr.085464.108 PMC3776646

[vbag207-B13] Huttenhower C , Gevers D, Knight R et al Structure, function and diversity of the healthy human microbiome. Nature 2012;486:207–14. 10.1038/nature11234 22699609 PMC3564958

[vbag207-B14] Johnson NL , Kotz S, Balakrishnan N. Continuous Univariate Distributions. Vol. 1, 2nd edn. New York: Wiley, 1994.

[vbag207-B15] Kang DD , Li F, Kirton E et al MetaBAT 2: an adaptive binning algorithm for robust and efficient genome reconstruction from metagenome assemblies. PeerJ 2019;7:e7359. 10.7717/peerj.7359 31388474 PMC6662567

[vbag207-B16] Karlin S. Assessing inhomogeneities in bacterial long genomic sequences. In: Waterman MS (ed), *Proceedings of the First Annual International Conference on Computational Molecular* Biology, Santa Fe, NM, USA. New York, NY: ACM Press, 1997, 164–71.

[vbag207-B17] Kellom M , Berg M, Chen I-MA et al Tetranucleotide frequencies differentiate genomic boundaries and metabolic strategies across environmental microbiomes. mSystems 2025;10:e01744-24. 40626735 10.1128/msystems.01744-24 PMC12363243

[vbag207-B18] Konishi S , Kitagawa G. Information Criteria and Statistical Modeling. New York: Springer, 2008.

[vbag207-B19] Li Y , Lyu H, Yang H et al FoodMicroDB: a microbiome database for composition and time-series research in food. iMetaOmics 2024;1:e40. 41676126 10.1002/imo2.40 PMC12806411

[vbag207-B20] Limpert E , Stahel WA, Abbt M. Log-normal distributions across the sciences: keys and clues. Bioscience 2001;51:341–52. 10.1641/0006-3568(2001)051[0341:LDATSK]2.0.CO;2

[vbag207-B21] Liu C-C , Dong S-S, Chen J-B et al MetaDecoder: a novel method for clustering metagenomic contigs to reconstruct microbial communities. Microbiome 2022;10:46. 10.1186/s40168-022-01237-8 35272700 PMC8908641

[vbag207-B22] Liu Y-X , Qin Y, Chen T et al A practical guide to amplicon and metagenomic analysis of microbiome data. Protein Cell 2021;12:315–30. 32394199 10.1007/s13238-020-00724-8 PMC8106563

[vbag207-B23] Mallawaarachchi V , Lin Y. Accurate binning of metagenomic contigs using composition, coverage, and assembly graphs. J Comput Biol 2022;29:1357–76. 10.1089/cmb.2022.0262 36367700

[vbag207-B24] Mallawaarachchi V , Wickramarachchi A, Xue H et al Solving genomic puzzles: computational methods for metagenomic binning. Brief Bioinform 2024;25:bbae372. 10.1093/bib/bbae372 39082646 PMC11289683

[vbag207-B25] Meyer F , Fritz A, Deng Z-L et al Critical assessment of metagenome interpretation: the second round of challenges. Nat Methods 2022;19:429–40. 10.1038/s41592-022-01431-4 35396482 PMC9007738

[vbag207-B26] Nayfach S , Rodriguez-Mueller B, Garud N et al An integrated metagenomics pipeline for strain profiling reveals novel patterns of bacterial transmission and biogeography. Genome Res 2016;26:1612–25. 27803195 10.1101/gr.201863.115 PMC5088602

[vbag207-B27] Nayfach S , Roux S, Seshadri R et al; IMG/M Data Consortium. A genomic catalog of earth’s microbiomes. Nat Biotechnol 2021;39:499–509. 33169036 10.1038/s41587-020-0718-6 PMC8041624

[vbag207-B28] Noble PA , Citek RW, Ogunseitan OA. Tetranucleotide frequencies in microbial genomes. Electrophoresis 1998;19:528–35. 9588798 10.1002/elps.1150190412

[vbag207-B29] Nurk S , Meleshko D, Korobeynikov A et al metaSPAdes: a new versatile metagenomic assembler. Genome Res 2017;27:824–34. 10.1101/gr.213959.116 28298430 PMC5411777

[vbag207-B30] Philippot L , Raaijmakers JM, Lemanceau P et al Going back to the roots: the microbial ecology of the rhizosphere. Nat Rev Microbiol 2013;11:789–99. 24056930 10.1038/nrmicro3109

[vbag207-B31] Pride DT , Meinersmann RJ, Wassenaar TM et al Evolutionary implications of microbial genome tetranucleotide frequency biases. Genome Res 2003;13:145–58. 10.1101/gr.335003 12566393 PMC420360

[vbag207-B32] Quince C , Walker AW, Simpson JT et al Shotgun metagenomics, from sampling to analysis. Nat Biotechnol 2017;35:833–44. 10.1038/nbt.3935 28898207

[vbag207-B33] Richardson L , Allen B, Baldi G et al MGnify: the microbiome sequence data analysis resource in 2023. Nucleic Acids Res 2023;51:D753–9. 36477304 10.1093/nar/gkac1080 PMC9825492

[vbag207-B34] Riesenfeld CS , Schloss PD, Handelsman J. Metagenomics: genomic analysis of microbial communities. Annu Rev Genet 2004;38:525–52. 15568985 10.1146/annurev.genet.38.072902.091216

[vbag207-B35] Roux S , Adriaenssens EM, Dutilh BE et al Minimum information about an uncultivated virus genome (MIUViG). Nat Biotechnol 2019;37:29–37. 30556814 10.1038/nbt.4306 PMC6871006

[vbag207-B36] Scher JU , Abramson SB. The microbiome and rheumatoid arthritis. Nat Rev Rheumatol 2011;7:569–78. 21862983 10.1038/nrrheum.2011.121 PMC3275101

[vbag207-B37] Schwarz G. Estimating the dimension of a model. Ann Statist 1978;6:461–4. 10.1214/aos/1176344136

[vbag207-B38] Sedlar K , Kupkova K, Provaznik I. Bioinformatics strategies for taxonomy independent binning and visualization of sequences in shotgun metagenomics. Comput Struct Biotechnol J 2017;15:48–55. 10.1016/j.csbj.2016.11.005 27980708 PMC5148923

[vbag207-B39] Sharpton TJ. An introduction to the analysis of shotgun metagenomic data. Front Plant Sci 2014;5:209. 24982662 10.3389/fpls.2014.00209 PMC4059276

[vbag207-B40] Simmons SL , DiBartolo G, Denef VJ et al Population genomic analysis of strain variation in leptospirillum group II bacteria involved in acid mine drainage formation. PLoS Biology 2008;6:e177. 18651792 10.1371/journal.pbio.0060177 PMC2475542

[vbag207-B41] Teeling H , Meyerdierks A, Bauer M et al Application of tetranucleotide frequencies for the assignment of genomic fragments. Environ Microbiol 2004b;6:938–47. 15305919 10.1111/j.1462-2920.2004.00624.x

[vbag207-B42] Teeling H , Waldmann J, Lombardot T et al TETRA: analysis and comparison of tetranucleotide usage patterns in DNA sequences. BMC Bioinformatics 2004a;5:163. 10.1186/1471-2105-5-163 15507136 PMC529438

[vbag207-B43] Venter JC , Remington K, Heidelberg JF et al Environmental genome shotgun sequencing of the sargasso sea. Science 2004;304:66–74. 10.1126/science.1093857 15001713

[vbag207-B44] Walter J , Ley R. The human gut microbiome: ecology and recent evolutionary changes. Annu Rev Microbiol 2011;65:411–29. 21682646 10.1146/annurev-micro-090110-102830

[vbag207-B45] Wang D , Li J, Su L et al Phylogenetic diversity of functional genes in deep-sea cold seeps: a novel perspective on metagenomics. Microbiome 2023;11:276. 38102689 10.1186/s40168-023-01723-7 PMC10722806

[vbag207-B46] Wasserstein RL , Lazar NA. The ASA’s statement on p-values: context, process, and purpose. Am Stat 2016;70:129–33. 10.1080/00031305.2016.1154108

[vbag207-B47] Weibull W. A statistical distribution function of wide applicability. J Appl Mech 1951;18:293–7.

[vbag207-B48] White H. Maximum likelihood estimation of misspecified models. Econometrica 1982;50:1–25. 10.2307/1912526

[vbag207-B49] Wu Y-W , Simmons BA, Singer SW. MaxBin 2.0: an automated binning algorithm to recover genomes from multiple metagenomic datasets. Bioinformatics 2016;32:605–7. 10.1093/bioinformatics/btv638 26515820

[vbag207-B50] Wu Y-W , Tang Y-H, Tringe SG et al MaxBin: an automated binning method to recover individual genomes from metagenomes. Microbiome 2014;2:26. 10.1186/2049-2618-2-26 25136443 PMC4129434

[vbag207-B51] Xia H , Zhang Z, Luo C et al MultiPrime: a reliable and efficient tool for targeted next-generation sequencing. iMeta 2023;2:e143. 38868227 10.1002/imt2.143 PMC10989836

